# Diagnostic Accuracy of Endobronchial Ultrasound-Guided Transbronchial Needle Aspiration (EBUS-TBNA) in Real Life

**DOI:** 10.3389/fmed.2020.00118

**Published:** 2020-04-07

**Authors:** Mukunthan Murthi, Elio Donna, Sixto Arias, Nestor R. Villamizar, Dao M. Nguyen, Gregory E. Holt, Mehdi S. Mirsaeidi

**Affiliations:** ^1^Department of Pulmonary and Critical Care, Miami VA Medical Center, University of Miami Miller School of Medicine, Miami, FL, United States; ^2^Department of Cardiothoracic Surgery, Miami VA Medical Center, University of Miami Miller School of Medicine, Miami, FL, United States

**Keywords:** EBUS-TBNA, NSCLC, bronchoscopy, mediastinoscopy, staging, accuracy

## Abstract

**Background:** EBUS-TBNA is an integral tool in the diagnosis and staging of lung cancer and other diseases involving mediastinal lymphadenopathy. Most studies attesting to the performance of EBUS-TBNA are prospective analyses performed under strict protocols. The objective of our study was to compare the accuracy of EBUS-TBNA to surgery in diagnosing hilar and mediastinal pathologies in a tertiary hospital, staffed by pulmonologists with and without formal interventional pulmonary training.

**Methods:** We retrospectively analyzed subjects who underwent EBUS-TBNA followed by a confirmatory surgical procedure from January 2012 to December 2018. The primary outcome was to evaluate the accuracy of EBUS-TBNA in the diagnosis of all mediastinal disease. Secondary analyses determined the accuracy of EBUS-TBNA in cancer, NSCLC, and non-malignant lesions individually.

**Results:** One hundred and forty-three subjects had an EBUS-TBNA procedure followed by surgery. EBUS-TBNA for all pathologies had an accuracy of 81.2% (CI 95% 73.8–87.4) and sensitivity of 55.1% (CI 95% 41.5–68.3). The accuracy and sensitivity of individual groups were: cancer (81.7, 48.8%), NSCLC (84, 48.3%), and non-malignancy (78.9, 60%). The NSCLC group had 15 false negatives and 5 (33.3%) of them were due to non-sampling of EBUS accessible nodes. Missed sampling led to a change in the final staging in 8.6% of NSCLC subjects.

**Conclusion:** The accuracy of EBUS-TBNA across all groups was comparable to those reported previously. However, the sensitivity was comparatively lower. This was primarily due to the large number of EBUS-TBNA accessible lymph nodes that were not sampled. This data highlights the need for guidelines outlining the best sampling approach and lymph node selection.

## Introduction

Lung cancer is the leading cause of cancer death in both men and women in the United States. More than 225,000 new diagnoses and 160,000 deaths due to lung cancer occur annually in the United States (1). In many subjects, Mediastinal lymph node evaluation is required to pathologically stage lung cancer in order to determine the appropriate subsequent treatment modalities, including the decision to offer potentially curative surgery. Other malignancies and benign pathologies like sarcoidosis (2) can also require mediastinal and hilar lymph node biopsies. Since its introduction for clinical use in 2002, Endobronchial Ultrasound- transbronchial needle aspiration [EBUS-TBNA] has been extensively utilized for the evaluation of mediastinal and hilar lymph nodes as well as endobronchial lesions (3, 4). Compared to mediastinoscopy, the gold standard for mediastinal LN sampling, EBUS-TBNA is less invasive, can be performed on an outpatient basis with moderate sedation and can be used to sample hilar lymph nodes (5).

As with any medical advancement, EBUS-TBNA has its own limitations. EBUS-TBNA cannot sample all the lymph nodes stations and the success of the technique depends on the provider's skills (6). However, EBUS-TBNA has consistently shown >95% accuracy in diagnosis and staging of lung cancer in multiple prospective protocol-based studies (7, 8). The accuracy of EBUS-TBNA in the detection of mediastinal metastasis from extra-thoracic malignancy and lymphoma was between 85–95% (9–12) and 91–97% (13), respectively. The EBUS-TBNA for the diagnosis of sarcoidosis had an accuracy of 79% (71–86) (14).

A drawback of these studies is that most of them report accuracy based on clinic-radiological follow-up without comparison to the gold standard of surgical biopsy, which might result in overestimation of accuracy. Studies on the real-life evaluation of the accuracy of the procedure by comparison with the surgical lymph node biopsy are limited. We aimed to evaluate the performance of EBUS-TBNA in diagnosing both malignant and benign lesions, at a tertiary hospital with bronchoscopists with and without formal interventional bronchoscopy training. We compared the results of EBUS-TBNA with those of mediastinoscopy or intrapleural surgery that evaluated mediastinal LNs.

## Methods

We conducted a retrospective, observational study analyzing all the subjects who underwent EBUS-TBNA of mediastinal and hilar lymph nodes followed by surgical intervention (mediastinoscopy and/or lymph node dissection) at the University of Miami Hospital (UMH) and the Miami Veterans Affairs (VA) medical center from first of January 2012 through end of December 2018. Pulmonologists at both hospitals are employed by the University of Miami.

In the VA setting, EBUS-TBNA is performed under general endotracheal anesthesia and in the University Hospital setting, the procedure is performed only under moderate sedation. All the EBUS-TBNA procedures were performed by pulmonologists.

EBUS-TBNA was performed using Olympus (BF-UC180F both at Miami VA and UMH) equipment. Selective sampling of lymph nodes by EBUS-TBNA was performed based on discussions between the pulmonologists and thoracic surgeons. If EBUS-TBNA was positive for mediastinal LN involvement, those cases were discussed at tumor board for consideration of induction therapy if single station and non-bulky (<3 cm) lymph nodes and considered for definitive chemotherapy-radiotherapy if multi-station or bulky lymph nodes. If EBUS-TBNA was negative, the same criteria as described above was used for selective mediastinal staging through mediastinoscopy before anatomic resection. Lymph node dissection of N1 and N2 was routinely performed in all surgeries for lung cancer.

EBUS-TBNA cytology was performed as previously reported (2). Surgical blocks were performed as standard procedure for surgical and EBUS tissue biopsies. Final reports of EBUS-TBNA and surgical biopsies were compared. Cytology and tissue slides were reviewed by staff pathologists located at each hospital separately.

All subjects who underwent EBUS-TBNA in both the centers were maintained in a separate directory. Medical records of each subject was reviewed to identify individuals who underwent surgical procedures following EBUS-TBNA. Only those who were subjected to subsequent surgical procedures were included in this study. Subjects who did not have a surgical procedure following EBUS-TBNA and subjects who did not have lymph nodes sampled during surgery or EBUS-TBNA were excluded from the study.

### Statistical Analysis

Descriptive variables, including demographics, EBUS-TBNA, and surgical procedures, were reported in numbers and percentages. We described the EBUS-TBNA test characteristics in comparison to surgery by calculating sensitivity and specificity and test relevance by calculating the positive predictive value (PPV) and negative predictive value (NPV). True positives (TP) were defined as LNs with the same pathological diagnosis in EBUS-TBNA and surgery and true negatives (TN) were defined as LNs without pathological findings in both EBUS-TBNA and surgery. False negatives (FN) were defined as either EBUS-TBNA finding an absence of disease in an LN but the presence of disease on surgical evaluation or EBUS failing to sample accessible pathological lymph node. False positives (FP) were defined as EBUS-TBNA finding disease that was not seen in the pathology from surgical specimens. Accuracy of EBUS-TBNA was defined as the ability to correctly diagnose the presence or absence of pathology in lymph nodes and calculated by an equation TP+TN/TP+TN+FP+FN. Our primary outcome was to define the accuracy of EBUS-TBNA in detecting any type of pathology in mediastinal and hilar lymph nodes. Secondary outcomes evaluated the accuracy of EBUS separately in the diagnosis of cancer, NSCLC and non-malignant lesions individually.

## Results

A total of 948 subjects underwent EBUS-TBNA during the study period, of which 143 had a subsequent surgical procedure. From the remaining 805, 218(27%) had negative EBUS-TBNA sampling of lymph nodes, 304 (37.7%) had NSCLC, 50 (6.2%) SCLC, and 117 (14.5%) had other malignancies (lymphomas, carcinoid, metastasis from extra-thoracic disease). One hundred and sixteen (14.4%) patients had non-cancer diagnosis. In the 143 subjects, 117(81.8%) had PET/CT, and 26(18.2%) had only CT before EBUS-TBNA procedure. Four subjects were not included in the final analysis as the lymph nodes identified with pathology on surgery were not accessible by EBUS-TBNA (Intraparenchymal nodes and stations 5, 6, 8, 9). The mean interval between EBUS-TBNA and the surgical procedure was 39 days (ranging from 2 to 142 days). The demographics of the study group are shown in the table below (Table 1).

**Table 1:** Demographics and procedure details.

Mean Age	67 (22–89)
**Sex**	
Male	87
Female	56
**Ethnicity**	
European-American	64
African-American	13
Latino	59
Asian	2
Other	5
**Number of Lymph nodes sampled Per EBUS**	
1	42
2	46
3	42
4	12
5	1
Total number of lymph nodes sampled	315
Average lymph node per EBUS	2.2

**Figure 1:**
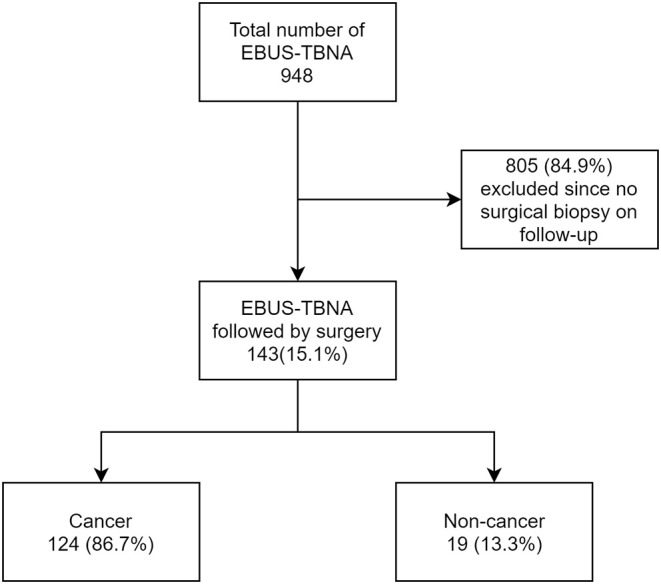
Flow chart showing breakdown of all EBUS cases.

Of the 139 subjects appropriate for analysis, 124 subjects (86.7%) had a primary diagnosis of cancer and 19 subjects (13.3%) had non-malignant lesions (Figure 1).

### Overall Accuracy of EBUS-TBNA

For all 139 subjects, our data found 32 true positives (23%), 26 false negatives (18.7%), and 81 (58.3%) true negatives. There were no false positives. Data analysis found the sensitivity was 55.1% (CI 95% 41.5–68.3), specificity was 100%, was PPV 100%, NPV was 75.7% (CI 95% 70–80.5) and accuracy was 81.2% (CI 95% 73.8–87.4).

### EBUS-TBNA in Detecting Cancer

**Figure 2:**
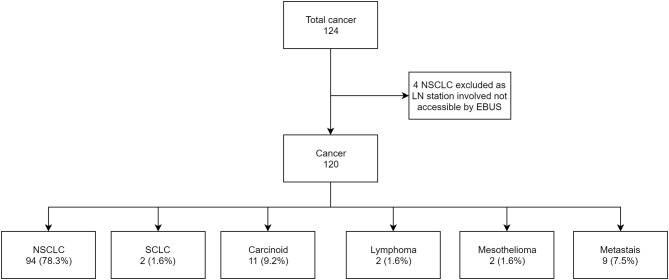
Flow chart showing cancer subtypes in subject who underwent EBUS-TBNA.

Of the 120 (86.3%) subjects with a diagnosis of cancer, the number of true positives was 21 (17.5%), false negative was 22 (18.3%) and true negative was 77 (64.2%). The sensitivity, specificity, PPV, NPV, and accuracy of EBUS-TBNA for cancer was 48.8% (CI 95% 33.3–64.5), 100%, 100%, 77.8% (CI 95% 72.3–82.4) and 81.7% (CI 95% 73.6–88.1), respectively (Figure 2). On further analysis of the 22 false negative lesions, we found 7 lymph nodes (32%) where surgical evaluation found malignancy in EBUS-TBNA accessible lymph nodes but were not sampled during bronchoscopy. The lymph node stations not sampled include 4L,4R and 7 (in 2 subjects), 10L,10R and 11R (in 2 subjects) and 12R in one subject.

### EBUS-TBNA in Detecting NSCLC

Out of 139 subjects, 94 (67.6%) had NSCLC as the primary diagnosis. Of the primary tumors, 33.3% originated in the right upper lobe (RUL), 21.9% in the left upper lobe (LUL), 10.4% in the right middle lobe (RML), 15.6% in the right lower lobe (RLL) and 18.7% in the left lower lobe (LLL). Data revealed 14 true positives (14.9%), 15 false negatives (15.9%), and 65 true negatives 65 (69.1%). Among the false negatives, 5/15 (33.3%) were due to non-sampling of EBUS accessible lymph nodes. Statistically, EBUS-TBNA for NSCLC had a sensitivity of 48.3% (CI 95% 29.5–67.5), specificity of 100%, PPV of 100%, NPV of 81.2% (CI 95% 75.3–86), and accuracy of 84% (CI 95% 75–90.8). The locations of the primary tumor were fairly evenly distributed in the false positives; 22% in RML, 11% in RLL, 16.7% in RML, 27.8% in LUL and 22% in LLL.

**Figure 3:**
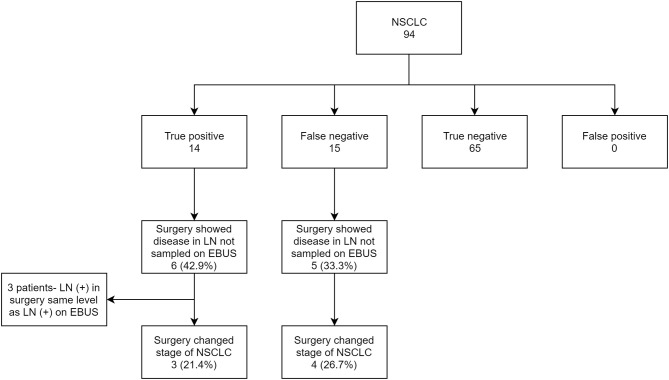
Flow chart showing EBUS-TBNA performance in NSCLC and comparison of EBUS staging with surgical staging.

Among true positive subjects, we further analyzed to identify the proportion of cases where EBUS-TBNA showed the highest station of lymph node involved in NSCLC. Out of 14 True positives, 10 showed the highest stage of NSCLC on EBUS. In 4 subjects, EBUS-TBNA showed involvement of only N1 station, but surgery showed involvement of N2 nodes (LN 7 in three subjects and LN 4R in one subject). Only 1 had the concerned N2 node punctured on EBUS. The other 3 did not have the involved N2 lymph node sampled (Figure 3).

### Effect of Missed Lymph-Node Sampling

With the number of lymph nodes not sampled in EBUS-TBNA in consideration, we further analyzed the effect of missed EBUS biopsy in both true positive and false negative subjects and compared the EBUS-TBNA and final surgery staging for NSCLC. In total, 11 NSCLC subjects had a surgical procedure diagnose disease in lymph nodes not sampled by EBUS-TBNA. In 3 subjects (27.3%), the involved stations in surgery was the same N staging compared to EBUS-TBNA and were excluded. In the remaining 8 subjects (72.7%), surgery showed newly diagnosed N1 or N2 disease compared to EBUS-TBNA. This resulted in a change of final staging in 7/8 (87.5%) of these subjects (Table 2).

**Table 2:** Effect of Lymph node not sampled on EBUS on staging.

**LN (+) on Surgery**	**LN seen on CT**	**Size**	**LN seen on PET**	**Size**	**Staging-EBUS**	**Staging- surgery**	**EBUS- surgery staging**
11L	N	-	N	-	T4N0M0	T4N1	3A to 3A
10L	Y	1.9 cm	N	-	T3N0M0	T3N1	2B to 3A
10R	Y	0.8 cm	Y-SUV-3.2	-	T1cN0	T1cN1	1A to 2B
7	Y	1 cm	N	-	T3N1	T3N2	3A to 3B
12R	NA		N	-	T2N0M0	T2N1M0	2A to 2B
7, 11R*	N	N	Y-LN 7-SUV-3.5, LN 11R-SUV 3.2	LN 7–1 cm	T2aN0	T2aN2	2A to 3A
4R	Y	1.4 cm	Y-SUV-3.9	NA	T2N1	T2N2	2B to 3A
7	NA	-	N	-	T3N1	T3N2	3A to 3B

### EBUS-TBNA in Detecting Non-cancer

Nineteen (13.7%) out of 139 subjects had non-malignant lesions on final diagnosis. 12 subjects (63.1%) had granulomas and the remaining had infection and nonspecific inflammation. There were 6 true positives (31.6%), 4 false negatives (21%), and 9 true negatives (47.4%). The performance of EBUS in non-malignant disease found a sensitivity of 60% (CI 95% 26.2–87.8), specificity of 100%, PPV of 100%, NPV of 69.2% (CI 95% 51.3–82.8), and an accuracy of 78.9% (CI 95% 54.4–93.9). The 4 false-negative LNs on EBUS-TBNA here were found to have granulomas on surgical pathology.

Among the 143 subjects, only 11 had core biopsies done in the EBUS procedure. Only 2 subjects had a pathological diagnosis in core biopsy which was negative in TBNA cytology, with one patient having mini-forceps biopsy of station 8 diagnostic of carcinoid and one patient having mini-forceps biopsy of station 12R diagnostic of metastatic renal cell carcinoma.

## Discussion

The results of our study show that EBUS-TBNA had an accuracy of 81.2% in the diagnosis of mediastinal and hilar lymph node pathology. Disease-specific accuracy was 81.7%, 84% and 78.9% in the diagnosis of cancer, NSCLC and non-cancer lesions, respectively. The accuracy achieved was largely comparable with those reported in literature (14–17). The sensitivities for all pathologies, cancer, NSCLC and non-cancer were 55.1, 48.8, 48.3, and 60%, respectively. The NPV for all pathologies, cancer, NSCLC and non-cancer were 75.7, 77.8, 81.2, and 69.2%, respectively.

The paramount utility of EBUS-TBNA is for the identification of lymph nodal metastasis in NSCLC, and the sensitivity of EBUS-TBNA in regards to this varies widely, with various studies reporting sensitivity ranging from 46 to 96% (18, 19). The sensitivity from our study across all groups was indeed lower than most reported in the literature.

One reason for such a result is the relatively high number of lymph nodes that were involved in pathology but were not sampled by EBUS-TBNA, leading to a false-negative result. In 8 subjects with NSCLC, EBUS-TBNA did not sample the lymph node that was positive on surgery. Apart from a high false-negative rate, the effect of missed lymph node sampling also resulted in surgical upstaging of cancer in 7 subjects with NSCLC. All of them had PET/CT before EBUS-TBNA but uptake in the metastatic lymph node was seen in only 3 of the subjects and CT showed enlarged lymph node larger than 1cm in 3 of them. In 3 subjects who had PET-positive lymph nodes, EBUS-TBNA sampled LN 11R, 8, 13R, and 2R, whereas surgery showed involvement of LN 10R, 7,12R and 4R, respectively. In one subject where CT alone identified enlarged infra-hilar lymph node of size 1.9 cm, EBUS-TBNA sampled 11L (negative) but surgery identified cancer in 10L.

The smallest lymph node metastasis identified on surgery was 8 mm in size on imaging. A systemic approach by the sampling of all mediastinal and hilar lymph nodes larger than 5 mm rather than a “targeted approach” whereby only abnormal lymph nodes seen on imaging are sampled, may result in fewer missed lymph nodes and thereby improved sensitivity. This approach has shown to reduce the number of subjects requiring upstaging after surgical procedure (20). Sampling of nodes in the order of N3 to N1 station is also recommended to avoid false diagnosis of higher stage of lung cancer (21). The major drawback is the prolonged procedure time (22).

Another reason for the low sensitivity of EBUS-TBNA in our study could be our rigorous study methodology. An analysis of six review articles/meta-analysis of EBUS showed that only 16.3% (7/43) were retrospective studies. All the others were prospective studies with strict protocols. Most of these studies report EBUS-TBNA performance based on clinico-radiological comparison and follow-up, rather than comparing it with pathology results from surgical sampling. Moreover, several studies attesting the accuracy of EBUS comes from a small group of authors, all interventional pulmonologists (18, 23–27). In the above mentioned six review articles describing the sensitivity of EBUS-TBNA, 43 individual studies were analyzed. The total number of authors was only 30, and 37% of the studies were from authors with multiple studies (16/43). This illustrates considerable expertise in the field. Hence, generalizing those results to the real world, especially in teaching and community hospitals where a substantial amount of training and learning period is involved, may not show the most accurate picture. One more aspect to consider is publication bias, as many authors who concur poor results from their studies hesitate to report them (28).

In the 4 subjects where lymph nodes were not accessible by EBUS-TBNA, one was intraparenchymal. The other nodes that were inaccessible were 5,6,8 and 9 the latter two of which can be reached using EUS-FNA (23). Studies have demonstrated that combined use of EBUS and EUS can improve sensitivity in detecting mediastinal metastasis (19). However, none of the subjects in our study underwent EUS.

The use of EBUS-TBNA in the diagnosis of mediastinal and hilar lymph node pathology has become more and more prevalent due to its lower cost, less invasiveness and fewer adverse events compared to surgical lymph node sampling. Combined with high accuracy, it makes for the ideal first step in the diagnosis of pathology in mediastinal and hilar lymph nodes. The main aim of our study was to identify the performance of EBUS-TBNA by comparing it with surgical pathology outcomes in a tertiary hospital setting where no protocols and guidelines of prospective studies are involved. This study highlights the fact that in centers where guidelines are not followed thoroughly, the outcomes are clearly inferior. We strongly encourage that hospitals and providers must follow the latest guidelines defined for selection and sampling of lymph nodes for EBUS-TBNA (21, 29, 30).

Our study has the limitations pertinent to retrospective studies. Some of the patients had an interval of more than 3 months between EBUS-TBNA and surgical confirmation due to unavoidable circumstances and this might have affected the results. Also, as with any endoscopic procedure, the probability of missing small foci of metastasis on EBUS-TBNA also needs to be considered.

## Conclusion

As EBUS-TBNA sampling of Mediastinal and hilar lymph node for pathological diagnosis and cancer staging becomes commonplace, more evaluation of its performance in conventional hospital settings is needed. Even though the performance of EBUS-TBNA in our center had a satisfactory accuracy of 81.2% for all pathologies, the low sensitivity was a cause for concern. This is especially imperative in lung cancer, where critical treatment decisions are made based on EBUS-TBNA results. Guidelines describing the best sampling approach and lymph node size threshold for needle puncture should be followed to achieve better outcomes.

## Data Availability Statement

All datasets generated for this study are included in the article/supplementary material.

## Ethics Statement

The studies involving human participants were reviewed and approved by University of Miami Institutional review board. Written informed consent for participation was not required for this study in accordance with the national legislation and the institutional requirements.

## Author Contributions

MM and MSM were involved in data collection, study design, analysis, preparation and review of manuscript. ED and SA were involved in data collection and review of manuscript. NV, DN, and GH were involved in review of manuscript.

### Conflict of Interest

The authors declare that the research was conducted in the absence of any commercial or financial relationships that could be construed as a potential conflict of interest.
